# Effect of APOE ε4 genotype on amyloid-β and tau accumulation in Alzheimer’s disease

**DOI:** 10.1186/s13195-020-00710-6

**Published:** 2020-10-31

**Authors:** Min Seok Baek, Hanna Cho, Hye Sun Lee, Jae Hoon Lee, Young Hoon Ryu, Chul Hyoung Lyoo

**Affiliations:** 1 Department of Neurology, Gangnam Severance Hospital, Yonsei University College of Medicine, 20 Eonjuro 63-gil, Gangnam-gu, Seoul, South Korea; 2 Biostatistics Collaboration Unit, Gangnam Severance Hospital, Yonsei University College of Medicine, Seoul, South Korea; 3 Department of Nuclear Medicine, Gangnam Severance Hospital, Yonsei University College of Medicine, 20 Eonjuro 63-gil, Gangnam-gu, Seoul, South Korea

**Keywords:** Alzheimer disease, Amyloid-β, ApoE, Positron emission tomography, Tau

## Abstract

**Background:**

To assess the effects of apolipoprotein E (ApoE) ε4 genotype on amyloid-β (Aβ) and tau burden and their longitudinal changes in Alzheimer’s disease (AD) spectrum.

**Methods:**

Among 272 individuals who underwent PET scans (^18^F-florbetaben for Aβ and ^18^F-flortaucipir for tau) and ApoE genotyping, 187 individuals completed 2-year follow-up PET scans. After correcting for the partial volume effect, we compared the standardized uptake value ratio (SUVR) for Aβ and tau burden between the ε4+ and ε4− groups. By using a linear mixed-effect model, we measured changes in SUVR in the ApoE ε4+ and ε4− groups.

**Results:**

The ε4+ group showed greater baseline Aβ burden in the diffuse cortical regions and greater tau burden in the lateral, and medial temporal, cingulate, and insula cortices. Tau accumulation rate was higher in the parietal, occipital, lateral, and medial temporal cortices in the ε4+ group. In Aβ+ individuals, baseline tau burden was greater in the medial temporal cortex, while Aβ burden was conversely greater in the ε4− group. Tau accumulation rate was higher in the ε4+ group in a small region in the lateral temporal cortex. The effect of ApoE ε4 on enhanced tau accumulation persisted even after adjusting for the global cortical Aβ burden.

**Conclusions:**

Progressive tau accumulation may be more prominent in ε4 carriers, particularly in the medial and lateral temporal cortices. ApoE ε4 allele has differential effects on the Aβ burden depending on the existing amyloidosis and may enhance vulnerability to progressive tau accumulation in the AD spectrum independent of Aβ.

**Supplementary information:**

**Supplementary information** accompanies this paper at 10.1186/s13195-020-00710-6.

## Background

Except for the rare dominantly inherited Alzheimer’s disease (AD), most AD patients are sporadic [1, 2]. The apolipoprotein E (ApoE) gene encodes a 35-kDa extracellular lipid and cholesterol carrier glycoprotein, and its ε4 allele is a major genetic risk factor for sporadic AD [1, 3]. The presence of this allele increases the risk of AD in a dose-dependent manner and lowers the age at onset [4, 5]. However, its effect on the regional accumulation rates of two major pathological proteins—amyloid-β (Aβ) and tau—remains unclear.

Greater amounts of Aβ burden were observed in ε4 carriers than in non-carriers in previous postmortem and ^11^C-Pittsburgh compound B (PIB) positron emission tomography (PET) studies [6–8]. Longitudinal change in Aβ burden was also greater in ε4 carriers than non-carriers in some previous studies [9–11], while another longitudinal study did not find this association [12].

Postmortem studies showed more frequent neurofibrillary tangle pathology in ε4 carriers in a dose-dependent manner [7], a greater tangle pathology in AD patients with ε4 homozygotes [13], and an association of the ε4 allele with tangle pathology in the presence of Aβ [14]. In contrast, another study did not find evidence for these associations [15]. A recent ^18^F-flortaucipir PET study demonstrated that ApoE ε4 had an Aβ-independent effect on the increase in the tau load in the entorhinal cortex and hippocampus [16], while the other studies found this effect was associated with the global Aβ burden [17] or even greater tau burden in the prodromal AD and AD dementia patients without the ε4 allele, particularly in the parietal cortex, than in patients who carried the ε4 allele [18].

In this study, we investigated the effects of the ε4 allele on regional Aβ and tau burden and their longitudinal changes in cognitively unimpaired (CU) individuals, mild cognitive impairment (MCI) patients, and AD patients.

## Materials and methods

### Participants

From January 2015 to August 2017, 272 individuals completed a baseline tau PET study at Gangnam Severance Hospital. The baseline study included magnetic resonance (MR) and two PET scan (^18^F-florbetaben for Aβ and ^18^F-flortaucipir for tau) studies, neuropsychological tests using Seoul Neuropsychological Screening Battery (tests for global cognition and six cognitive domains) [19], and ApoE genotyping. In 187 individuals who agreed to a follow-up study, the same neuroimaging and neuropsychological tests were performed after a mean of 2.0 ± 0.3 years.

We used the clinical diagnostic criteria for probable AD dementia proposed by the National Institute of Neurological and Communicative Disorders and Stroke, and used the Alzheimer’s Disease and Related Disorders Association and Petersen’s criteria for diagnosing MCI [20, 21]. Accordingly, the baseline study included 96 CU, 105 MCI, and 71 AD dementia patients, and the longitudinal study included 80 CU, 42 MCI, and 65 AD dementia patients. Baseline Aβ-positivity was determined by two nuclear medicine specialists using the validated visual assessment methods [22, 23]. Detailed information for the inclusion of participants has been described in our previous reports [24, 25].

### Acquisition of PET and MR images

We performed ^18^F-florbetaben and ^18^F-flortaucipir PET in separate days, almost within a month (8.3 ± 7.9 days for the baseline and 9.4 ± 7.6 days for the follow-up scans). PET images were acquired in a Biograph mCT PET/CT scanner (Siemens Medical Solutions; Malvern, PA, USA) for 20 min at 90 min after the injection of ^18^F-florbetaben and at 80 min after the injection of ^18^F-flortaucipir. Prior to the scan, brain computed tomography images were acquired for attenuation correction. 3D PET images were reconstructed using the ordered-subsets expectation maximization (OSEM) algorithm in a 256 × 256 × 223 matrix with a 1.591 × 1.591 × 1 mm voxel size. MR images were scanned within 90 days before or after the acquisition of ^18^F-flortaucipir PET (27.7 ± 25.7 days for the baseline and 13.4 ± 18.0 days for the follow-up scans). In a 3.0-T MR scanner (Discovery MR750, GE Medical Systems, Milwaukee, WI), T1-weighted MR images were acquired using 3D-spoiled gradient-recalled sequences (repetition time = 8.3 ms, echo time = 3.3 ms, flip angle = 12°, 512 × 512 matrix, voxel spacing 0.43 × 0.43 × 1 mm).

### Image processing steps

We used FreeSurfer 5.3 (Massachusetts General Hospital, Harvard Medical School; http://surfer.nmr.mgh.harvard.edu) software to obtain participant-specific volumes-of-interest (VOIs). In brief, T1-weighted MR images were resliced to a 1-mm isovoxel space in FreeSurfer, corrected for inhomogeneity, and segmented into gray and white matter. After tessellation, 3D surfaces for gray and white matter were reconstructed. Subcortical regions were segmented with the probabilistic registration method [26], and cortical regions were probabilistically labeled based on the curvature information overlaid on an inflated white matter surface [27, 28]. Finally, participant-specific composite VOI images, including 16 and 4 subcortical regions, were created by merging anatomically related regions. Detailed list of VOIs and their corresponding regions in the Desikan-Killiany atlas was presented in Table S1.

Statistical parametric mapping 12 (Wellcome Trust Centre for Neuroimaging, London, UK) and in-house software implemented in MATLAB 2015b (MathWorks, Natick, MA, USA) were used for the processing of PET images. PET images were first coregistered to MR images that had been resliced to the FreeSurfer isovoxel space. Using participant-specific composite VOI images, PET images were corrected for partial volume effect (PVE) with a region-based voxel-wise (RBV) method [29]. The standardized uptake value ratio (SUVR) images were created using the cerebellar crus median obtained by overlaying a template mask on PET images spatially that were normalized with diffeomorphic anatomical registration through an exponentiated lie algebra tool [30]. Finally, regional SUVR values were obtained by overlaying the participant-specific composite VOI images on individual PET images.

For visualization, cortical uptake values were mapped on the white matter surface by assigning the values of voxels corresponding to the mid-point between the gray and white matter surface, corrected for PVE with the RBV method, and then converted to SUVR maps using the cerebellar crus median as a reference. Surface SUVR images were spatially normalized and finally smoothed on a 2D surface using a Gaussian kernel with 8-mm full-width half-maximum.

### Statistical analysis

SPSS 23 (IBM Corp., Armonk, NY, USA) was used for the statistical analysis of demographic data and baseline VOI data. Continuous and categorical demographic variables were compared between the ApoE ε4− and ε4+ groups using independent *t* test and chi-square test, respectively. Using the general linear model with age, years of education, sex, and baseline Mini-Mental State Examination (MMSE) scores as covariates, the baseline SUVR values were compared between the ApoE ε4− and ε4+ groups. *P* values for trends were calculated using analysis of covariance (ANCOVA) after adjusting for age, sex, years of education, and baseline MMSE scores as covariates. We included MMSE scores as a covariate in all statistical analysis due to the difference in the distribution of cognitive status between the ε4+ and ε4− groups. Region-wise multiple comparisons were corrected for using Benjamini-Hochberg’s false discovery rate (FDR) method (FDR-corrected *P* < 0.05 for 17 regions) [31]. Likewise, baseline surface images were compared between the two groups using the same general linear model implemented in FreeSurfer. Longitudinal changes in the regional SUVR values and surface images were compared between the groups using a linear mixed-effect model in MATLAB with age, sex, years of education, baseline MMSE scores, and an interaction term between the presence of ApoE ε4 and time intervals as fixed factors, under the assumption of a random intercept and slope, by setting the intervals and subject as random factors.

We primarily analyzed data with four covariates above and repeated the analysis with the baseline global cortical Aβ burden as an additional covariate.

## Results

### Demographic characteristics

Baseline and follow-up demographic data are summarized in Table 1 and S2. In individuals included in the baseline and follow-up studies, age, sex, and education did not differ between the ε4− and ε4+ groups. The ε4+ group showed higher proportions of Aβ-positivity and clinical dementia, and worse global cognition at baseline than the ε4− group. However, none of the demographic characteristics and global cognition differed between each groups stratified by Aβ-positivity. Compared to baseline, global cognition had worsened at follow-up in both the ε4− and ε4+ groups. The number of ε4 carriers was greater in patients with dementia than that in CU and MCI patients.

**Table 1:** Baseline demographic characteristics of 272 participants who completed the baseline study

	Aβ±		Aβ−		Aβ+	
	ε4−	ε4+	ε4−	ε4+	ε4−	ε4+
***N***	195	77	134	24	61	53
**Baseline age (years)**	70.4 ± 10.3	70.0 ± 8.6	68.4 ± 10.3	65.5 ± 8.3	74.7 ± 8.7	72.1 ± 7.9
**Females (%)**	127 (65%)	51 (66%)	90 (67%)	16 (67%)	37 (61%)	35 (66%)
**Education (years)**	11.1 ± 4.9	11.2 ± 5.0	11.0 ± 4.9	11.4 ± 4.2	11.2 ± 4.8	11.1 ± 5.3
**Duration (years)**	2.6 ± 1.5	3.1 ± 1.4	2.3 ± 1.5	2.4 ± 1.3	3.0 ± 1.5	3.2 ± 1.4
**Aβ-positivity (%)**	61 (31%)	53 (69%)^a^	0 (0%)	0 (0%)	61 (100%)	53 (100%)
**ε2/2:ε2/3:ε3/3**	1:37:157	n.a.	1:31:102	n.a.	0:6:55	n.a.
**ε2/4:ε3/4:ε4/4**	n.a.	2:60:15	n.a.	2:21:1	n.a.	0:39:14
**Baseline diagnosis, CU/MCI/DEM (%)**	79/75/41 (41/38/21%)	17/30/30^a^ (22/39/39%)	72/49/13 (54/37/10%)	15/7/2 (63/29/8%)	7/26/28 (11/43/46%)	2/23/28 (4/43/53%)
**MMSE**	25.4 ± 4.7	23.5 ± 5.3^a^	26.8 ± 3.2	26.7 ± 2.5	22.2 ± 5.7	22.1 ± 5.7
**CDR-SB**	1.6 ± 2.3	2.7 ± 2.5^a^	0.9 ± 1.5	0.8 ± 1.4	3.1 ± 3.0	3.6 ± 2.4

Data are presented as mean ± SD

*Abbreviations*: *CU* cognitively unimpaired, *MCI* mild cognitive impairment, *DEM* dementia, *Aβ±* Aβ-positivity, *ApoE* apolipoprotein E, *MMSE* Mini-Mental State Examination, *CDR-SB* Clinical Dementia Rating sum-of-boxes

^a^*P* < 0.05 for the comparisons between the ε4− and ε4+

### Baseline Aβ and tau burden

**Fig. 1:**
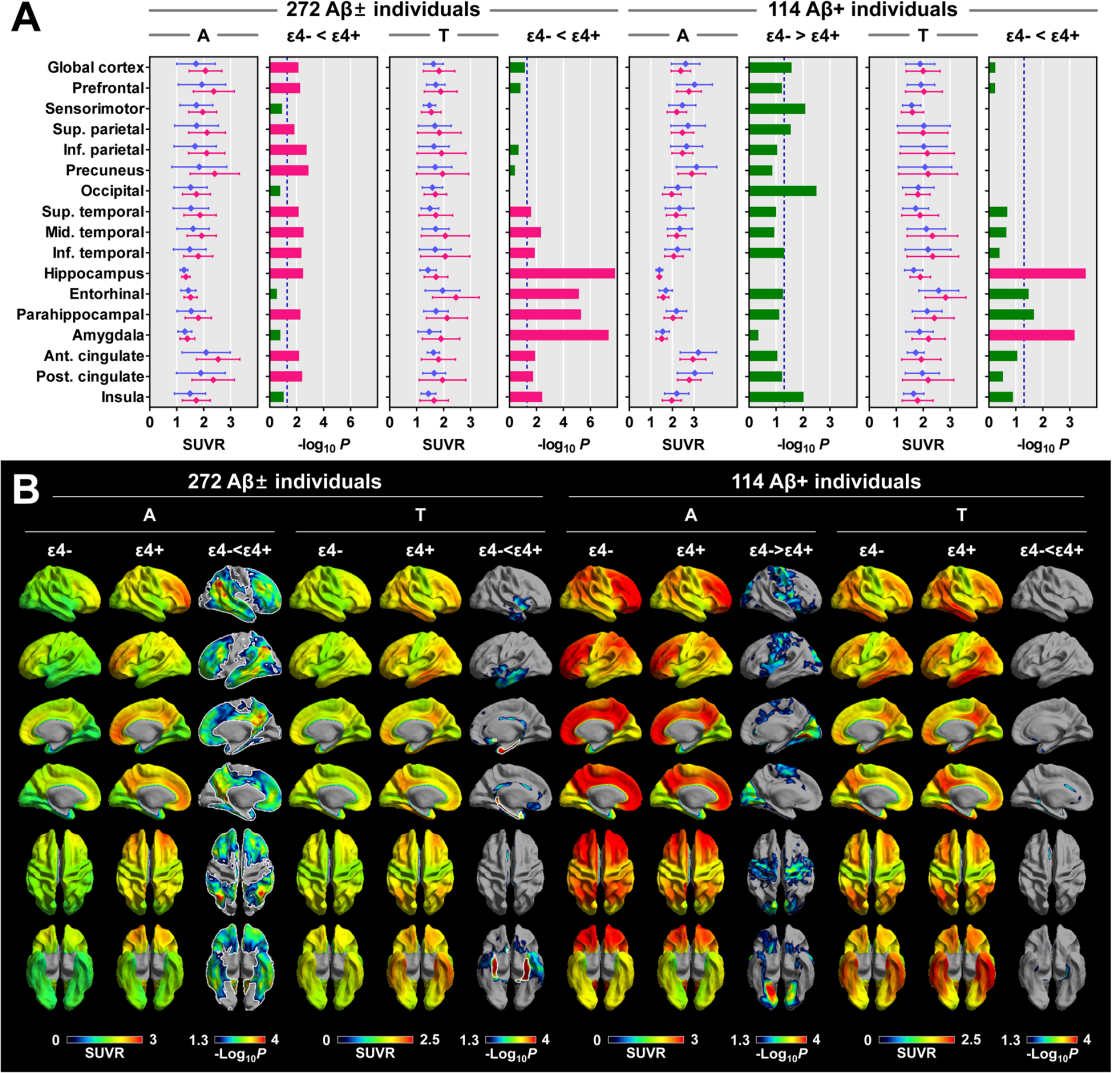
Comparison of baseline ^18^F-florbetaben and ^18^F-flortaucipir SUVR between the ApoE ε4− and ε4+ groups. **a** VOI-based comparisons between the ApoE ε4− and ε4+ groups. Data are presented as means (dots) and standard deviations (error bars) of the ε4− (blue) and ε4+ (red) groups. *P* values for the comparison between the ε4− and ε4+ groups are expressed as -Log_10_*P*. Red bars represent the regions that survived correcting for region-wise multiple comparisons (false discovery rate-corrected *P* < 0.05), and blue dotted lines represent uncorrected *P* = 0.05. **b** Surface-based comparisons between the ApoE ε4− and ε4+ groups. Regions surrounded by white lines (ε4− < ε4+ in Aβ and tau burden in all individuals) represent the cortical areas that survived correcting for multiple comparisons (false discovery rate-corrected *P* < 0.05). *P* values for the comparison between the ε4− and ε4+ groups are expressed as -Log_10_*P*. Aβ±, Aβ-positivity; ApoE, apolipoprotein E; SUVR, standardized uptake value ratio; A, ^18^F-florbetaben; T, ^18^F-flortaucipir

In all 272 Aβ− and Aβ+ individuals, the ApoE ε4+ group exhibited greater Aβ burden in the global cortex; prefrontal, parietal, lateral temporal, parahippocampal, and cingulate cortices; and hippocampus than the ε4− group, and all regions survived correcting for multiple comparisons. Conversely, in 114 Aβ+ individuals, Aβ burden was greater in the ε4− group in the global cortex, and sensorimotor, superior parietal, occipital, and insula cortices than in the ε4+ group, although all regions did not survive correcting for multiple comparisons (Fig. 1a). Surface-based statistics showed similar results as VOI-based comparisons (Fig. 1b).

In all individuals, greater tau burden was observed in the ε4+ group in the lateral and medial temporal, cingulate, and insula cortices, and all regions survived multiple comparisons (Fig. 1a). In Aβ+ individuals, the ε4+ group showed greater tau burden in the medial temporal regions, of which only the amygdala and hippocampus survived correcting for multiple comparisons. Surface-based statistics showed greater tau burden in the medial temporal and anterior cingulate regions in the ε4+ group than in the ε4− group; however, none of the regions survived after correcting for multiple comparisons (Fig. 1b).

Increased baseline Aβ burden in the hippocampus was associated with the number of ε4 alleles. Likewise, tau burden in the medial temporal regions showed an association with ε4 allele in a dose-dependent manner (Fig. 5a). In Aβ+ individuals, increased tau burden in the hippocampus and amygdala was associated with the ε4 allele in a dose-dependent manner (Fig. 5b).

**Fig. 2:**
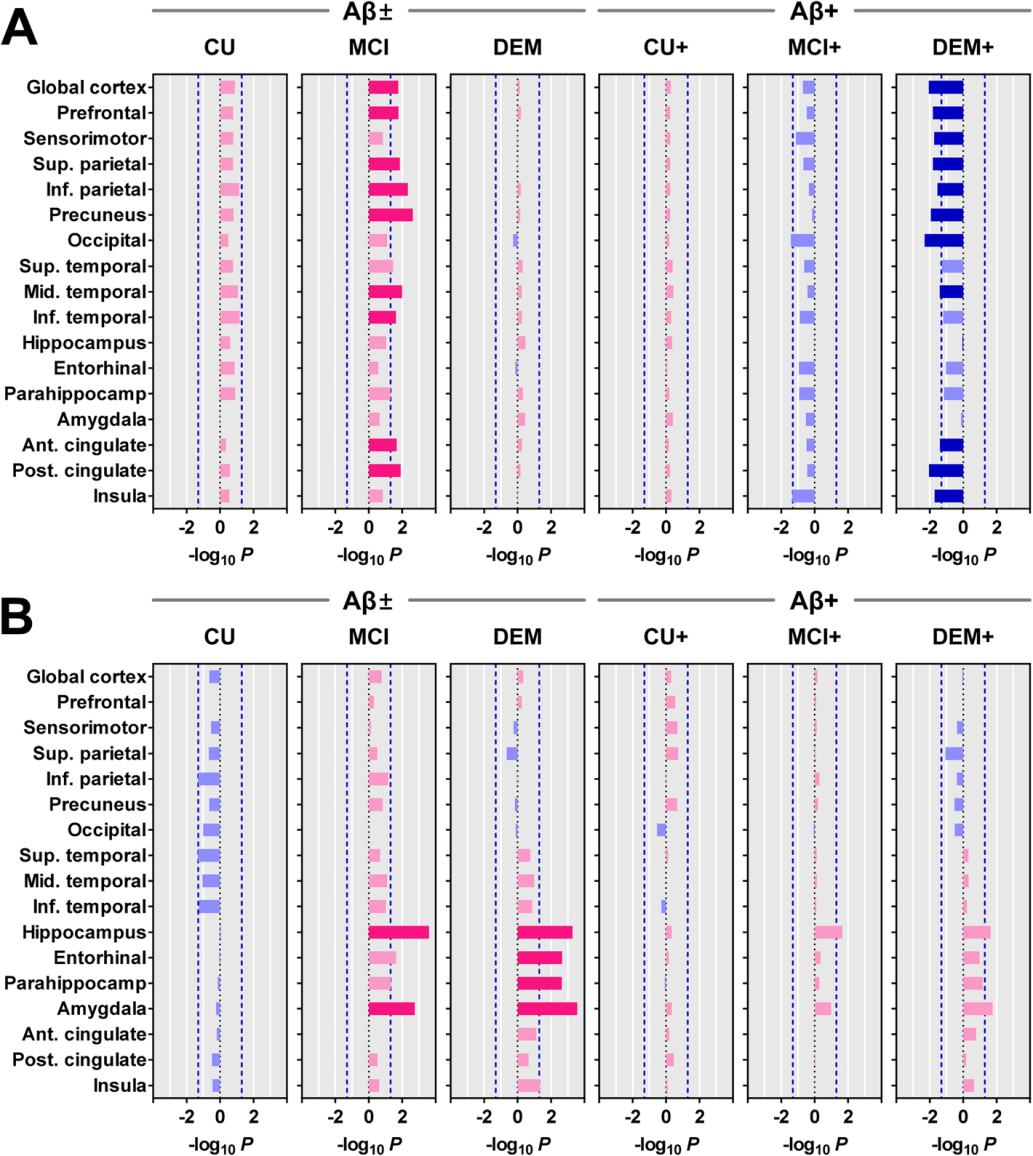
Comparison of baseline ^18^F-florbetaben (**a**) and ^18^F-flortaucipir (**b**) SUVR values between the ApoE ε4− and ε4+ groups in each group for cognitive status. Blue and light blue bars represent the -Log_10_*P* for ApoE ε4− > ε4+, and red and light red bars represent the -Log_10_*P* for ApoE ε4− < ε4+. Blue and red bars represent the regions that survived after correcting for multiple comparisons (false discovery rate-corrected *P* < 0.05), and blue dotted lines represent uncorrected *P* = 0.05

We also compared baseline SUVR values between the two ApoE groups within each group for cognitive status (Fig. 2). When compared to the ε4− group, tau burden was greater in the ε4+ group in the hippocampus in Aβ+ MCI patients and in the hippocampus and amygdala in Aβ+ AD dementia patients. However, all regions did not survive correction for multiple comparisons.

When the baseline global cortical Aβ burden was included as an additional covariate in the model, the ApoE ε4+ group exhibited greater tau burden in all medial temporal regions when compared to the ε4− group (Additional file 1: Fig. S2A).

### Longitudinal changes in Aβ and tau burden

**Fig. 3:**
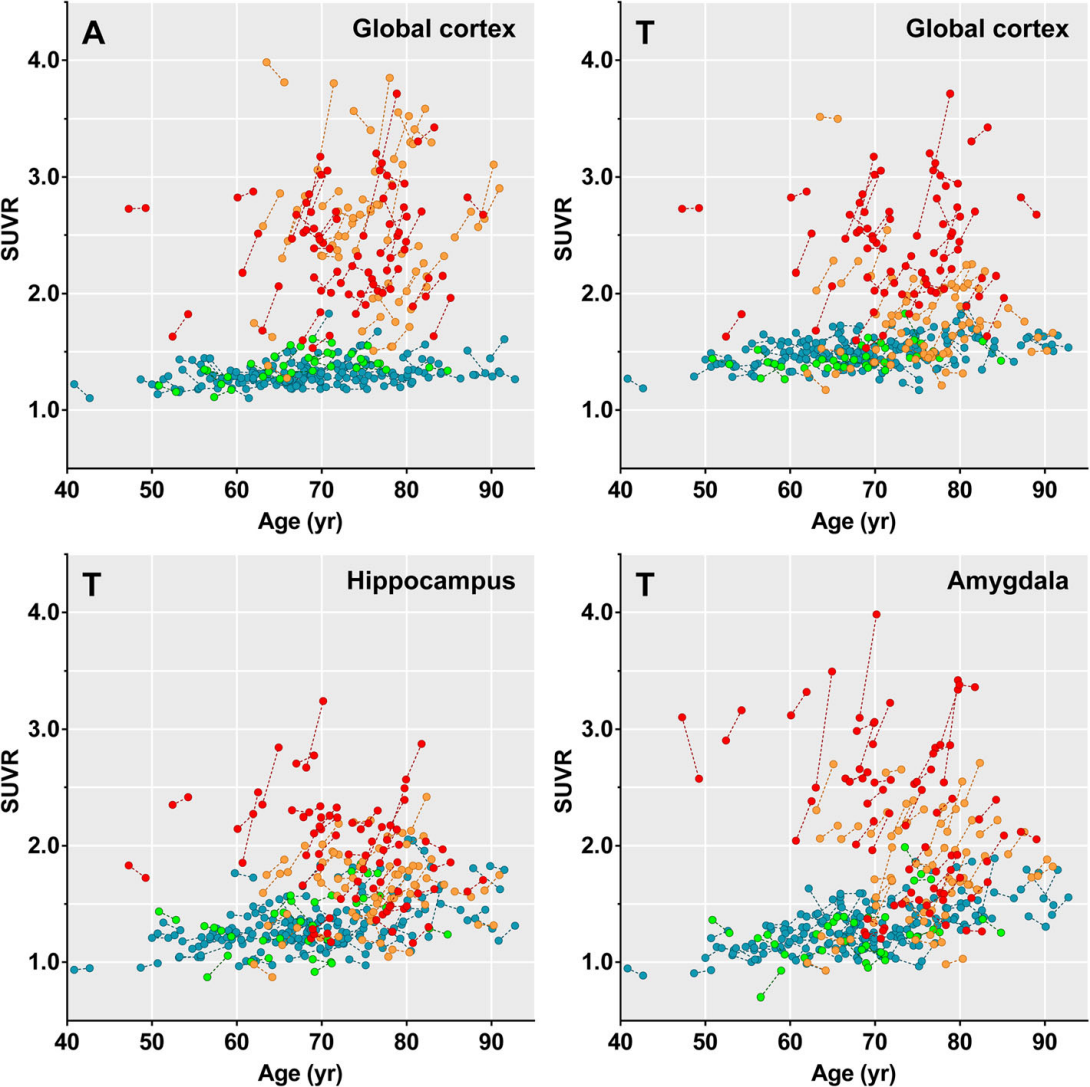
Spaghetti plots showing individual changes in regional SUVR values. In 187 Aβ± individuals, the individual data points measured at baseline and follow-up are displayed as color-coded dots (cyan = Aβ−/ApoE ε4−, green = Aβ−/ApoE ε4+, orange = Aβ+/ApoE ε4−, red = Aβ+/ApoE ε4+). Aβ±, Aβ-positivity; ApoE, apolipoprotein E; SUVR, standardized uptake value ratio; A, ^18^F-florbetaben; T, ^18^F-flortaucipir

**Fig. 4:**
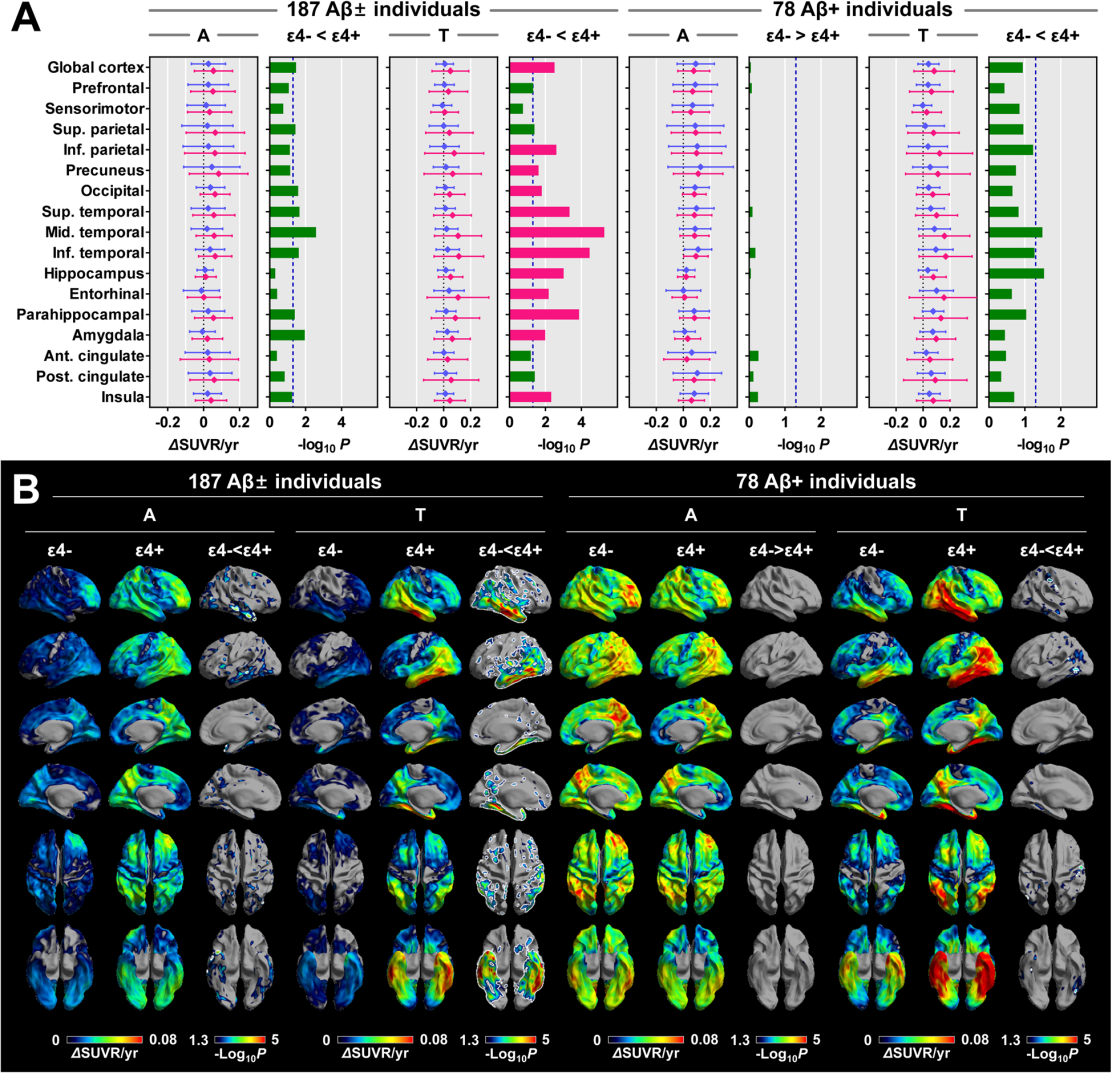
Comparison of annual changes in ^18^F-florbetaben and ^18^F-flortaucipir SUVR between the ApoE ε4− and ε4+ groups. **a** VOI-based comparison between the ApoE ε4− and ε4+ groups. Data are presented as means (dots) and standard deviations (error bars) of the ε4− (blue) and ε4+ (red) groups. *P* values for the comparison between the ε4− and ε4+ groups are expressed as -Log_10_*P*. Red bars represent the regions that survived correcting for region-wise multiple comparisons (false discovery rate-corrected *P* < 0.05), and blue dotted lines represent uncorrected *P* = 0.05. **b** Surface-based comparisons between the ApoE ε4− and ε4+ groups. Regions surrounded by white lines (ε4− < ε4+ in Aβ and tau accumulation rates in all individuals, and ε4− < ε4+ in tau accumulation rate in Aβ+ individuals) represent the cortical areas that survived correcting for multiple comparisons (false discovery rate-corrected *P* < 0.05). *P* values for the comparison between the ε4− and ε4+ groups are expressed as -Log_10_*P*. *P* values for the comparison between the baseline and follow-up are expressed as -Log_10_*P*. Aβ±, Aβ-positivity; ApoE, apolipoprotein E; SUVR, standardized uptake value ratio; A, ^18^F-florbetaben; T, ^18^F-flortaucipir

Examples of baseline Aβ and tau burden and their changes at follow-up are demonstrated in Fig. 3. In all 187 individuals, the ε4+ group exhibited a higher Aβ accumulation rate than the ε4− group in the global cortex; superior parietal, occipital, lateral temporal, and parahippocampal cortices; and amygdala; however, none of the regions survived correcting for multiple comparisons (Fig. 4a). A surface-based comparison showed a higher Aβ accumulation rate in diffuse cortical areas in the ε4+ group than in the ε4− group, and small regions in the lateral temporal cortex survived correcting for multiple comparisons (Fig. 4b). In Aβ+ individuals, there was no difference in the Aβ accumulation rate between the two groups.

**Fig. 5:**
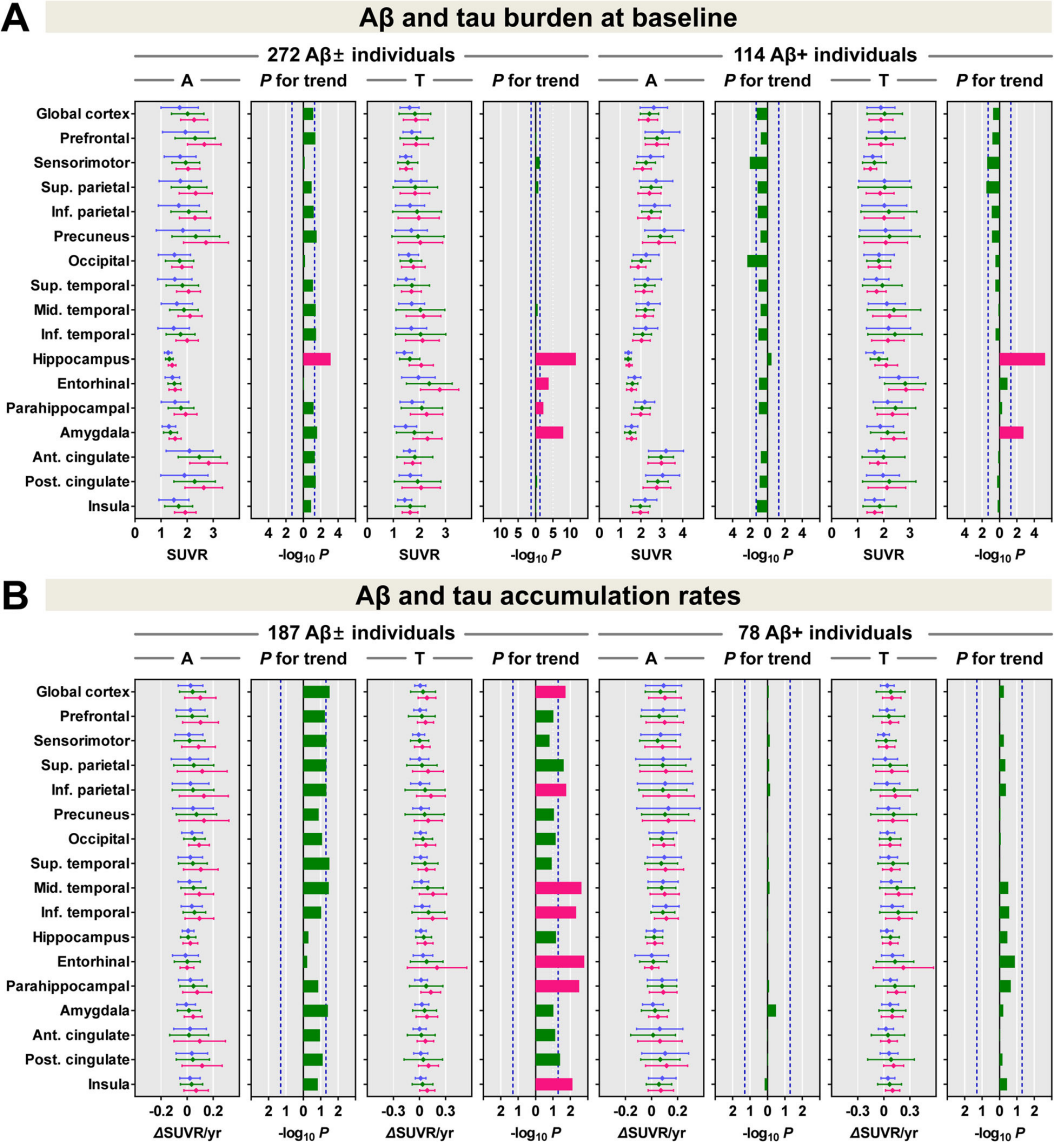
*P* for trend analysis of baseline ^18^F-florbetaben and ^18^F-flortaucipir SUVR and their longitudinal accumulation rates across the ApoE ε4-negative, heterozygous, and homozygous groups. Data are presented as means and standard deviations (error bars) of ε4-negative (blue), ε4-heterozygous (green), and ε4-homogygous (red) groups. Regions that showed significant differences in a dose-dependent manner after adjusting for sex, age, duration of education, and MMSE score (uncorrected *P* for trend < 0.05) and additionally survived correcting for region-wise multiple comparisons (false discovery rate-corrected *P* < 0.05) are presented as red bars. Blue dotted lines represent uncorrected *P* for trend = 0.05. Rightward direction of horizontal bars represents an SUVR value increased with higher numbers of ε4 alleles, while the leftward direction represents an SUVR value decreased with higher numbers of ε4 alleles. Aβ±, Aβ-positivity; ApoE, apolipoprotein E; SUVR, standardized uptake value ratio; A, ^18^F-florbetaben; T, ^18^F-flortaucipir

In all individuals, the tau accumulation rate in the ε4+ group was higher in the global cortex, and prefrontal, parietal, occipital, lateral and medial temporal, posterior cingulate, and insula cortices compared to the ε4− group. Except for the prefrontal, superior parietal, and posterior cingulate cortices, all regions survived correcting for multiple comparisons (Fig. 4a). Moreover, the increase in tau accumulation rate is associated with the number of ApoE ε4 allele (Fig. 5b). Similar to the VOI-based results, a surface-based comparison of the annual increase in tau showed a higher tau accumulation rate, particularly in the diffuse parietotemporal cortex in the ε4+ group (Fig. 4b). In Aβ+ individuals, the ε4+ group exhibited a greater annual increase in tau burden in the middle temporal and hippocampus, although none of the regions survived correcting for multiple comparisons (Fig. 4a). Surface-based statistics also showed a higher tau accumulation rate in small regions in the basal and lateral temporal and sensorimotor cortices even after correction for multiple comparisons (Fig. 4b).

Even after inclusion of the baseline global cortical Aβ burden as an additional covariate in the model, the results for the VOI-based comparison of tau accumulation rates between the two ApoE groups were almost similar (Additional file 1: Fig. S2B).

## Discussion

In this study, we assessed the effects of the ApoE ε4 genotype on Aβ and tau burden and found a greater baseline Aβ and tau burden and higher tau accumulation rate in the ε4+ group than in the ε4− group. The Aβ accumulation rate in the ε4+ group was higher in small areas in the lateral temporal cortex. In Aβ+ individuals, the baseline tau burden in the ε4+ group was greater in the medial temporal regions and the tau accumulation rate in the ε4+ group was higher in small regions in the basal and lateral temporal cortices than in the ε4− group.

A transgenic mouse model with neuron-specific overexpression of ApoE ε4 showed greater phosphorylated tau burden in the neocortex and hippocampus [32], and tau transgenic mice expressing human ApoE ε4 exhibited greater tau burden in the hippocampus than those expressing ε2 or ε3 [33]. A postmortem study showed that ε4 gene dose-dependently increased neurofibrillary tangle (NFT) pathology, and there was greater NFT pathology in diffuse cortical areas in AD patients carrying the ε4 allele than in those who did not [7]. Another study showed greater cortical NFT pathology only in the AD patients homozygous for the ε4 allele than in those with a single ε4 allele or those without the allele [13]. Unlike these transgenic mice and human postmortem studies, human cerebrospinal fluid (CSF) biomarker studies showed no differences in the level of CSF T-tau and P-tau between the ε4+ and ε4− groups [34, 35]. Moreover, one cross-sectional ^18^F-flortaucipir PET study in Aβ+ MCI and AD patients demonstrated that the ε4− group conversely exhibited greater tau burden in the parieto-occipital cortex than the ε4+ group [18]. In our Aβ+ AD dementia patients, the ε4− group tended to show greater tau burden in the parieto-occipital cortex, similar to the previous study, while the ε4+ group tended to show greater tau burden in the medial temporal cortex. However, none of these regions survived correction for multiple comparisons (Fig. 2b). This discrepancy may be attributable to the disproportionate frequency of the ε4 allele in patients with different subtypes of AD. The hippocampal sparing type of AD is associated with a younger age at onset, lower frequency of the ApoE ε4 allele, greater tau burden particularly in the parietal cortex, faster cortical atrophy, and faster cognitive decline than the typical AD subtype [36–39]. Therefore, we suspect that inclusion of a greater proportion of the hippocampal sparing subtype in the study cohort diluted the effect of ε4 on the tau burden or may even have caused contrary results.

Although a previous report has demonstrated a longitudinal increase in CSF tau in AD patients [40], one longitudinal ^18^F-flortaucipir PET study performed in a small number of AD patients did not find an association between the ApoE genotypes and longitudinal changes in tau burden [41]. In our results for all Aβ± individuals, the regional tau accumulation rate was higher in diffuse regions in the medial and lateral temporal and parieto-occipital cortices in the ε4+ group than in the ε4− group. Moreover, even in Aβ+ individuals, a higher tau accumulation rate was observed in the ε4+ group in small regions in the temporal cortex, suggesting that the ApoE ε4 genotype had an effect on progressive tau accumulation.

One recent ^18^F-flortaucipir PET study including 325 individuals (90% cognitively unimpaired and 10% cognitively impaired) showed an association of ApoE ε4 with increased tau burden in the entorhinal cortex, but statistical significance was lost after adjusting for global cortical Aβ burden [17]. In contrast, another study that included 489 individuals with a more balanced distribution of cognitive status (57% cognitively unimpaired and 43% cognitively impaired) demonstrated that ApoE ε4 had an effect on the increased tau burden in the entorhinal cortex and hippocampus, and which persisted even after adjusting for global cortical Aβ burden, as we found in our study [16]. Moreover, the effect of ApoE ε4 on progressive tau accumulation was replicated after adjusting for global cortical Aβ burden in our longitudinal study. To evaluate the proportion of a direct effect of ApoE ε4+ for increasing regional tau burden, we additionally performed path analysis with the ApoE ε4 positivity as a predictor and global cortical Aβ burden as a mediator. There was a significant direct effect of ApoE ε4 on baseline tau burden in the medial temporal regions, and 49–66% of total effect was explained by direct effect. Likewise, a significant direct effect of ApoE ε4 on progressive tau accumulation in longitudinal study was observed in the lateral temporal and parahippocampal cortices and hippocampus, and 64–71% of total effect was explained by direct effect (Additional file 1: Table S3). Therefore, tau accumulation may be accelerated in the presence of ApoE ε4 independent of Aβ burden.

The ApoE ε4 isoform was more likely to stimulate neuronal Aβ production than the other isoforms in vitro [42], and transgenic mice expressing the ApoE ε4 isoform showed less effective clearance of soluble Aβ from brain interstitial fluid [43]. Human autopsy findings demonstrated greater Aβ burden in the ε4+ than in the ε4− group not only in AD patients [13], but also in the MCI patients and CU individuals [44]. Likewise, when compared to individuals without the ε4 allele, a greater Aβ burden was observed in the global cortex in CU individuals and in MCI patients with the ε4 allele [8], and in the temporo-parietal cortex in AD patients with ε4 allele based on the PET studies [45]. Our study also demonstrated greater Aβ burden in the diffuse cortical areas in individuals with the ε4 allele than in those without. In contrast to the strong association between the ε4 allele and the baseline Aβ burden, we found a weak effect of ApoE ε4 on progressive Aβ accumulation in small regions in the lateral temporal cortex only in all Aβ± individuals. The Aβ accumulation rate in Aβ+ individuals did not differ between the ε4+ and ε4− groups like previous studies [9, 11], suggesting an effect of the ApoE ε4 allele on Aβ deposition only in the early stage of the disease.

Interestingly, Aβ burden in the Aβ+ individuals was paradoxically greater in the ε4− group than in the ε4+ group, similar to previous ^11^C-PIB and ^18^F-fluorodeoxyglucose PET studies that demonstrated lower Aβ burden and contrarily greater cortical hypometabolism in the AD patients carrying the ε4 allele than in those without this allele [46, 47]. This paradoxical effect of the ApoE ε4 allele on Aβ deposition can be expected by clinical studies that found an impact of the ApoE ε4 allele on Aβ burden in CU and MCI but not in those with AD [8, 34]. Furthermore, a study with transgenic mice demonstrated enhanced Aβ aggregation by ApoE ε4 in the early seeding stage but not in the later Aβ growth stage [48]. An in vitro experiment demonstrated that ApoE ε4 binds to toxic Aβ oligomers and more potently delays further aggregation of Aβ into the PET-detectable fibril form than the other ApoE isoforms [49]. Therefore, ApoE ε4 may play an important role in Aβ accumulation in the early stages of AD pathogenesis rather than in the advanced stages and may be more likely to be exposed to toxic oligomers. Subsequently, events toward final neurodegeneration may be induced, thereby shifting the hypothetical biomarker curves for tau and neurodegeneration to the Aβ curve [47]. It is also interesting to note that a transgenic mice model expressing both Aβ and tau exhibited a smaller number of plaques than a model expressing only Aβ [50]. Greater microgliosis and reduction of the amyloid-precursor protein-producing neurons due to greater tau accumulation in ε4 carriers may be another possible mechanism underlying the paradoxically lower Aβ burden [50]. However, this hypothesis cannot fully explain the mechanism due to the mismatch between the cortical areas with greater Aβ burden in the ε4− group and those with greater tau burden in the ε4+ group (Fig. 1).

## Limitations

Our study showed greater tau burden in the medial temporal areas in all Aβ+ individuals carrying the ε4 allele than in those not carrying the ε4 allele, but the result for the hippocampus was limited by the off-target binding in the choroid plexus adjacent to the hippocampus. In addition, distribution of diagnoses was different between the ε4+ and ε4− groups, with global cognition being more impaired in the ε4+ group (Table 1). Consequently, we had to adjust for global cognition additionally in the group comparison to minimize the effect of differences in disease severity between groups. Another methodological limitation was the high variability and bias in the quantitation of longitudinal PET study with simple ratio method due to changes in perfusion [51]. Finally, a longer follow-up duration will be necessary to observe greater differences in progressive tau accumulation between the ε4+ and ε4− groups.

## Conclusions

Our study revealed that progressive tau accumulation may occur more prominently in ε4 carriers, particularly in the medial and lateral temporal cortices. The presence of the ε4 allele not only has differential effects on Aβ burden depending on the existing amyloidosis but also possibly enhances vulnerability to progressive tau accumulation in the AD spectrum independent of Aβ.

## Supplementary Information

**Additional file 1: Table S1.** Desikan-Killiany atlas and custom composite VOIs. **Table S2.** Diagnosis and status of ApoE ε4 genotype of participants. **Table S3.** Total and direct effect of ApoE ε4 on the baseline tau and annual changes in tau burden. **Fig. S1.** Comparison of baseline and annual changes in ^18^F-florbetaben and ^18^F-flortaucipir SUVR values uncorrected for partial volume effect between the ApoE ε4- and ε4+ groups. **Fig. S2.** Comparison of baseline ^18^F-flortaucipir SUVR values (A) and their changes at follow-up (B) between the ApoE ε4- and ε4+ groups after adjusting for the baseline Aβ burden.

## Data Availability

Data generated by this study are available from the corresponding author on reasonable request. The data are not publicly available due to privacy restriction.

## Abbreviations

ApoE: Apolipoprotein E
CU: Cognitively unimpaired
MCI: Mild cognitive impairment
NFT: Neurofibrillary tangle
PVE: Partial volume effect
SUVR: Standardized uptake value ratio
VOIs: Volumes-of-interest
