# Supplementary material for: Effect of APOE ε4 genotype on amyloid-β and tau accumulation in Alzheimer’s disease
Source: Alzheimers Res Ther. 2020 Oct 31;12:140. doi: 10.1186/s13195-020-00710-6 (PMC7603688; doi:10.1186/s13195-020-00710-6)
Supplement: Supplementary file 1 — Additional file 1: Table S1. Desikan-Killiany atlas and custom composite VOIs. Table S2. Diagnosis and status of ApoE ε4 genotype of participants. Table S3. Total and direct effect of ApoE ε4 on the baseline tau and annual changes in tau burden. Fig. S1. Comparison of baseline and annual changes in 18F-florbetaben and 18F-flortaucipir SUVR values uncorrected for partial volume effect between the ApoE ε4- and ε4+ groups. Fig. S2. Comparison of baseline 18F-flortaucipir SUVR values (A) and their changes at follow-up (B) between the ApoE ε4- and ε4+ groups after adjusting for the baseline Aβ burden. [file 13195_2020_710_MOESM1_ESM.doc]

**Table S1.** Desikan-Killiany atlas and custom composite VOIs

| **Desikan-Killiany atlas** | **Custom composite VOI** |
| --- | --- |
| frontal pole | Prefrontal |
| lateral orbitofrontal |
| medial orbitofrontal |
| pars opercularis |
| pars orbitalis |
| pars triangularis |
| rostral middle frontal |
| caudal middle frontal |
| superior frontal |
| paracentral | sensorimotor |
| precentral |
| postcentral |
| superior parietal | superior parietal |
| inferior parietal | inferior parietal |
| supramarginal |
| precuneus | precuneus |
| cuneus | occipital |
| pericalcarine |
| lateral occipital |
| lingual |
| bankssts | superior temporal |
| superior temporal |
| transverse temporal |
| temporal pole | middle temporal |
| middle temporal |
| inferior temporal | inferior temporal |
| fusiform |
| entorhinal | entorhinal |
| parahippocampal | parahippocampal |
| rostral anterior cingulate | anterior cingulate |
| caudal anterior cingulate |
| isthmus cingulate | posterior cingulate |
| posterior cingulate |
| insula | insula |

**Table S2.** Diagnosis and status of ApoE ε4 genotype of participants

|  | **CU** | **MCI** | **DEM** |
| --- | --- | --- | --- |
| **n** | 96 | 105 | 71 |
| **age (years)** | 66.3 ± 9.5 | 71.1 ± 9.1^a^ | 74.4 ± 9.6^a^ |
| **Gender (M : F)** | 36:60 | 41:64 | 17:54^b^ |
| **Education (years)** | 11.9 ± 4.5 | 11.2 ± 4.5 | 9.8 ± 5.7 |
| **MMSE** | 28.2 ± 1.8 | 25.7 ± 2.7^a^ | 19.1 ± 5.3^a,b^ |
| **CDR-SB** | 0 | 1.5 ± 1.0^a^ | 5.0 ± 2.5^a,b^ |
| **Amyloid positivity** | 9/87 (9%) | 49/58 (47%)^a^ | 56/15 (79%)^a,b^ |
| **ApoE ε4 allele** |  |  |  |
| **ε4 Negative(%)** | 79 (82%) | 75 (71%) | 41 (58%) |
| **ε4 Heterozygous(%)** | 16 (17%) | 22 (21%) | 24 (34%) |
| **ε4 Homozygous(%)** | 1 (1%) | 8 (8%) | 6 (9%) |

Data are presented as mean ± SD. ^a^*P* < 0.05 for the comparisons between the CU and each group. ^b^*P* < 0.05 for the comparisons between the MCI and DEM groups.

Abbreviations: CU = cognitively unimpaired; MCI = mild cognitive impairment; DEM = dementia; Aβ+/- = Aβ-positivity; ApoE = apolipoprotein-E; MMSE = Mini-Mental State Examination; CDR-SB = Clinical Dementia Rating sum-of-boxes

**Table S3. Total and direct effect of ApoE ε4 on the baseline tau and annual changes in tau burden**

|  | **Baseline ^18^F-flortaucipir SUVR** | | | | | | |
| --- | --- | --- | --- | --- | --- | --- | --- |
|  | **Total effect** | | |  | **Direct effect** | | |
|  | **β** | **95% CI** | ***P*-value** |  | **β** | **95% CI** | ***P*-value** |
| **Global cortex** | **0.208** | 0.075~0.344 | < 0.001 |  | 0.077 | -0.040~0.204 | 0.224 |
| **Prefrontal** | **0.183** | 0.053~0.330 | 0.004 |  | 0.073 | -0.057~0.208 | 0.242 |
| **Sensorimotor** | 0.065 | -0.017~0.155 | 0.118 |  | 0.008 | -0.068~0.086 | 0.852 |
| **Sup. parietal** | 0.166 | -0.031~0.386 | 0.112 |  | -0.007 | -0.208~0.197 | 0.934 |
| **Inf. parietal** | **0.282** | 0.077~0.502 | 0.006 |  | 0.090 | -0.100~0.281 | 0.366 |
| **Precuneus** | **0.275** | 0.057~0.533 | 0.012 |  | 0.079 | -0.132~0.304 | 0.474 |
| **Occipital** | **0.106** | 0.002~0.219 | 0.048 |  | 0.000 | -0.103~0.107 | 0.996 |
| **Sup. temporal** | **0.218** | 0.074~0.368 | 0.002 |  | 0.104 | -0.020~0.240 | 0.130 |
| **Mid. temporal** | **0.360** | 0.160~0.581 | < 0.001 |  | 0.165 | -0.007~0.367 | 0.056 |
| **Inf. temporal** | **0.372** | 0.159~0.591 | 0.002 |  | 0.142 | -0.043~0.336 | 0.148 |
| **Hippocampus** | **0.294** | 0.194~0.400 | < 0.001 |  | **0.195** | 0.109~0.293 | < 0.001 |
| **Entorhinal** | **0.495** | 0.276~0.708 | < 0.001 |  | **0.241** | 0.068~0.410 | 0.004 |
| **Parahippocampal** | **0.411** | 0.237~0.587 | < 0.001 |  | **0.216** | 0.076~0.376 | < 0.001 |
| **Amygdala** | **0.434** | 0.273~0.604 | < 0.001 |  | **0.258** | 0.129~0.390 | < 0.001 |
| **Ant. cingulate** | **0.186** | 0.055~0.342 | 0.004 |  | 0.127 | -0.003~0.283 | 0.058 |
| **Post. cingulate** | **0.309** | 0.111~0.536 | < 0.001 |  | 0.144 | -0.024~0.346 | 0.098 |
| **Insula** | **0.206** | 0.086~0.334 | 0.002 |  | 0.106 | -0.002~0.227 | 0.052 |
|  | **Annual change in ^18^F-flortaucipir SUVR** | | | | | | |
|  | **Total effect** | | |  | **Direct effect** | | |
|  | **β** | **95% CI** | ***P*-value** |  | **β** | **95% CI** | ***P*-value** |
| **Global cortex** | **0.042** | 0.011~0.080 | 0.010 |  | 0.026 | -0.006~0.069 | 0.106 |
| **Prefrontal** | 0.030 | -0.005~0.072 | 0.116 |  | 0.015 | -0.021~0.057 | 0.470 |
| **Sensorimotor** | 0.017 | -0.011~0.046 | 0.270 |  | 0.009 | -0.019~0.039 | 0.536 |
| **Sup. parietal** | 0.043 | -0.002~0.091 | 0.064 |  | 0.031 | -0.018~0.085 | 0.244 |
| **Inf. parietal** | **0.071** | 0.020~0.132 | 0.006 |  | 0.055 | -0.001~0.124 | 0.060 |
| **Precuneus** | 0.050 | -0.003~0.109 | 0.066 |  | 0.032 | -0.022~0.096 | 0.312 |
| **Occipital** | **0.033** | 0.003~0.065 | 0.032 |  | 0.017 | -0.012~0.048 | 0.270 |
| **Sup. temporal** | **0.053** | 0.021~0.095 | < 0.001 |  | **0.034** | 0.000~0.080 | 0.046 |
| **Mid. temporal** | **0.085** | 0.040~0.132 | < 0.001 |  | **0.056** | 0.012~0.103 | 0.012 |
| **Inf. temporal** | **0.083** | 0.037~0.135 | < 0.001 |  | **0.056** | 0.007~0.110 | 0.022 |
| **Hippocampus** | **0.035** | 0.011~0.064 | 0.004 |  | **0.025** | 0.002~0.052 | 0.044 |
| **Entorhinal** | **0.067** | 0.009~0.133 | 0.022 |  | 0.040 | -0.010~0.097 | 0.124 |
| **Parahippocampal** | **0.070** | 0.020~0.125 | 0.002 |  | **0.046** | 0.001~0.101 | 0.042 |
| **Amygdala** | **0.036** | 0.002~0.074 | 0.046 |  | 0.017 | -0.017~0.055 | 0.334 |
| **Ant. cingulate** | 0.029 | -0.007~0.071 | 0.138 |  | 0.020 | -0.017~0.066 | 0.342 |
| **Post. cingulate** | 0.042 | -0.009~0.105 | 0.118 |  | 0.023 | -0.028~0.090 | 0.394 |
| **Insula** | **0.034** | 0.006~0.063 | 0.014 |  | 0.019 | -0.009~0.050 | 0.196 |

Path analysis was modeled with an effect of ApoE ε4 on the regional tau burden indirectly mediated by global cortical Aβ burden (ApoE ε4 → global cortical Aβ → regional tau) and a direct effect of ApoE ε4 on the regional tau burden (ApoE ε4 → regional tau). Indirect effect of ApoE ε4 is a product of β-coefficients for ApoE ε4 to global cortical Aβ and global cortical Aβ to regional tau. Confidence intervals for the β-coefficients were calculated with 1,000 repetitions of bootstrapping. Regional β-coefficients with significant effect on tau burden are presented in bold numbers.

Abbreviation: CI = confidence interval

**
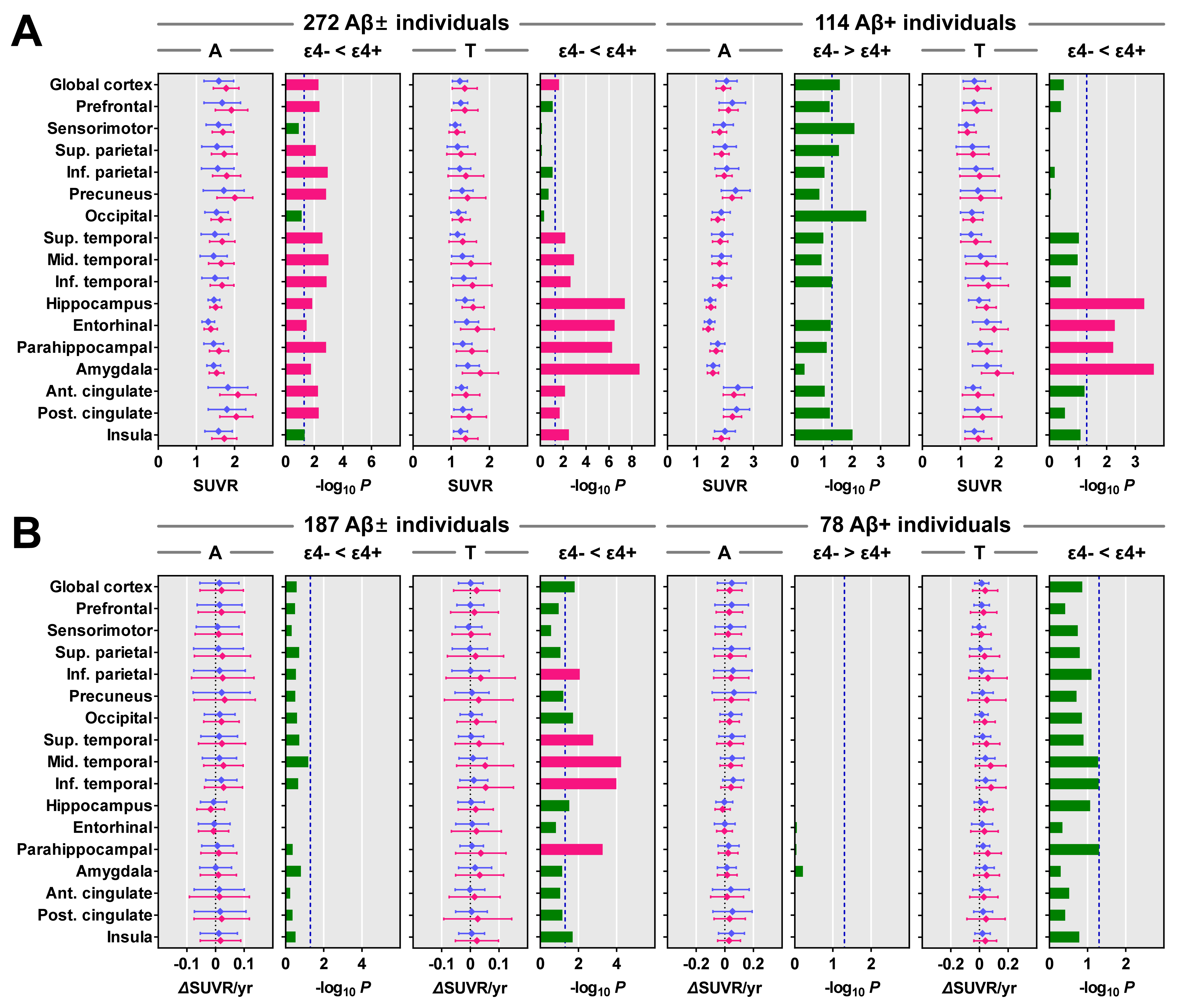
**

**Fig. S1.** Comparison of baseline and annual changes in ^18^F-florbetaben and ^18^F-flortaucipir SUVR values uncorrected for partial volume effect between the ApoE ε4- and ε4+ groups.

VOI-based comparisons of baseline (A) and annual changes (B) in SUVR between the ApoE ε4- and ε4+ groups. Data are presented as means (dots) and standard deviations (error bars) of the ε4- (blue) and ε4+ (red) groups. *P*-values for the comparison between the ε4- and ε4+ groups are expressed as -Log_10_*P*. Red bars represent the regions that survived correcting for region-wise multiple comparisons (false discovery rate-corrected *P* < 0.05), and blue dotted lines represent uncorrected *P* = 0.05.

Abbreviations: Aβ+/- = Aβ-positivity, ApoE = apolipoprotein-E, SUVR = standardized uptake value ratio, A = ^18^F-florbetaben, T = ^18^F-flortaucipir


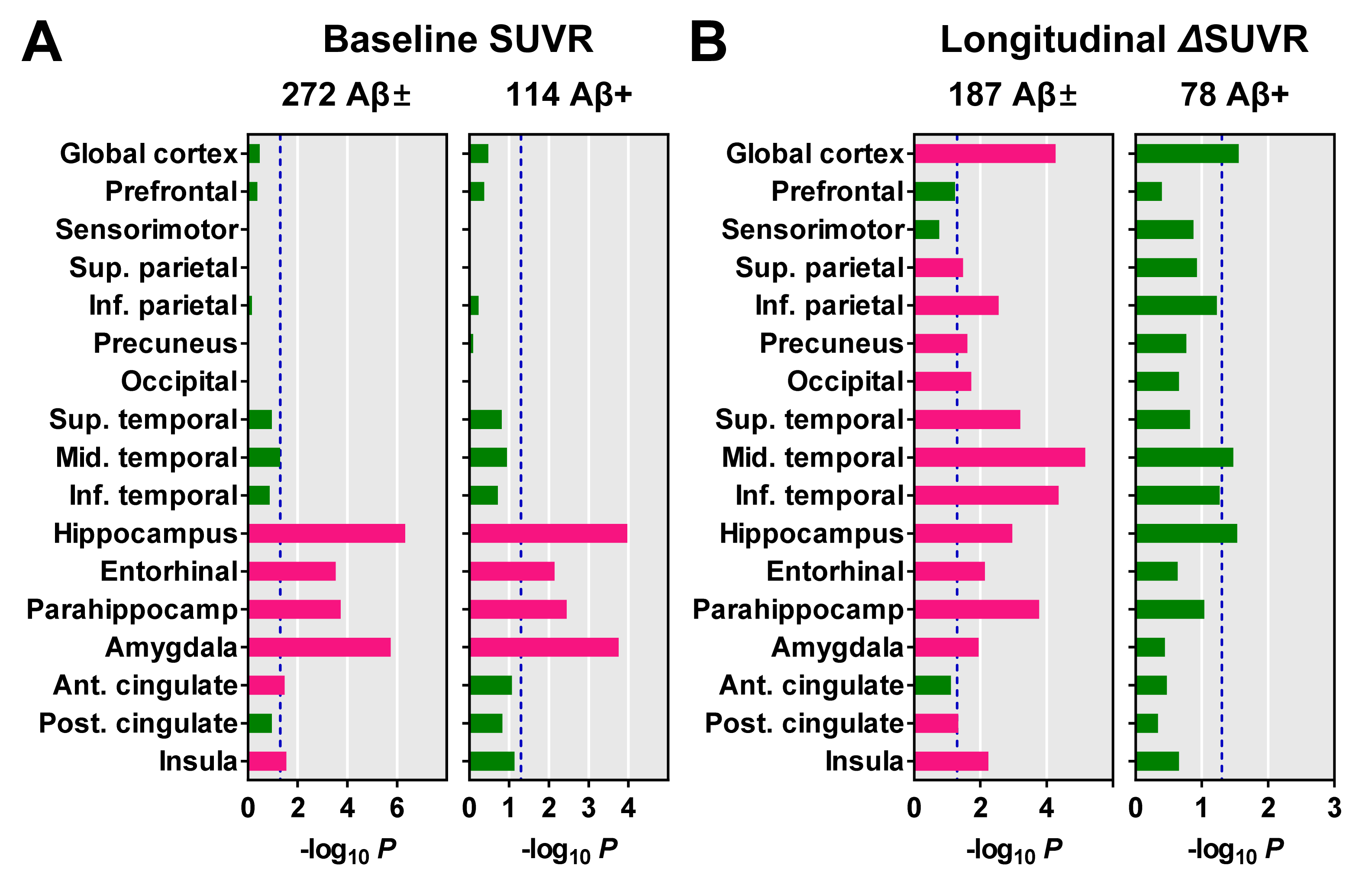


**Fig. S2.** Comparison of baseline ^18^F-flortaucipir SUVR values (A) and their changes at follow-up (B) between the ApoE ε4- and ε4+ groups after adjusting for the baseline Aβ burden

Red bars represent the regions that survived after correcting for multiple comparisons (false discovery rate-corrected *P* < 0.05), and blue dotted lines represent uncorrected *P* = 0.05.

Abbreviations: Aβ+/- = Aβ-positivity, ApoE = apolipoprotein-E, SUVR = standardized uptake value ratio
